# Red Blood Cell Distribution Width (RDW) as a Predictor of In-Hospital Mortality in COVID-19 Patients; a Cross Sectional Study

**DOI:** 10.22037/aaem.v9i1.1325

**Published:** 2021-10-13

**Authors:** Setareh Jandaghian, Atefeh Vaezi, Amirreza Manteghinejad, Maryam Nasirian, Golnaz Vaseghi, Shaghayegh Haghjooy Javanmard

**Affiliations:** 1 Applied Physiology Research Center, Cardiovascular Research Institute, Isfahan University of Medical Sciences, Isfahan, Iran.; 2 Cancer Prevention Research Center, Omid Hospital, Isfahan University of Medical Sciences, Isfahan, Iran.; 3 Epidemiology and Biostatistics Department, Health School, Infectious Diseases and Tropical Medicine Research Center, Isfahan University of Medical Sciences, Isfahan, Iran.; 4 Isfahan Cardiovascular Research Center, Cardiovascular Research Institute, Isfahan University of Medical Sciences, Isfahan, Iran.; * Setareh Jandaghian and Atefeh Vaezi are co-first authors.

**Keywords:** COVID-19, SARS-CoV-2, Erythrocyte Indices, Severity of Illness Index, Mortality

## Abstract

**Introduction:**

Red blood cell distribution width (RDW) has been introduced as a predictive factor for mortality in several critical illnesses and infectious diseases. This study aimed to assess the possible relationship between RDW on admission and COVID-19 in-hospital mortality.

**Method:**

This cross-sectional study was performed using the Isfahan COVID-19 registry. Adult confirmed cases of COVID-19 admitted to four hospitals affiliated with Isfahan University of Medical Sciences in Iran were included. Age, sex, O2 saturation, RDW on admission, Intensive Care Unit admission, laboratory data, history of comorbidities, and hospital outcome were extracted from the registry. Cox proportional hazard regression was used to study the independent association of RDW with mortality.

**Results:**

4152 patients with the mean age of 61.1 ± 16.97 years were included (56.2% male). 597 (14.4%) cases were admitted to intensive care unit (ICU) and 477 (11.5%) cases died. The mortality rate of patients with normal and elevated RDW was 7.8% and 21.2%, respectively (OR= 3.1, 95%CI: 2.6-3.8), which remained statistically significant after adjusting for age, O2 saturation, comorbidities, and ICU admission (2.03, 95% CI: 1.68-2.44). Moreover, elevated RDW mortality Hazard Ratio in patients who were not admitted to ICU was higher than ICU-admitted patients (3.10, 95% CI: 2.35-4.09 vs. 1.47, 95% CI: 1.15-1.88, respectively).

**Conclusion:**

The results support the presence of an association between elevated RDW and mortality in patients with COVID-19, especially those who were not admitted to ICU. It seems that elevated RDW can be used as a predictor of mortality in COVID-19 cases.

## 1. Introduction

The pandemic of coronavirus disease 2019 (COVID‐19) caused by Severe Acute Respiratory Syndrome Coronavirus 2 (SARS-CoV-2) is the greatest challenge of the current century. As reported by WHO, by August 2021, COVID‐19 has infected more than 215 million cases and caused more than 4 million deaths leading to enormous demands for healthcare services worldwide (1). Therefore, rapid, accurate, and early clinical assessment of the disease severity is vital. COVID-19 would start with lung involvement in some cases, which could quickly turn to acute respiratory distress syndrome (ARDS) and multi-systemic involvement, including the hematopoietic system (2, 3). The extraordinary case fatality rate of 59% for severe cases (4) indicates the need for identifying accessible laboratory markers for risk stratification and effective utilization of Intensive Care Unit (ICU) services.

Hematological abnormalities, which have been reported in patients with COVID-19, are considered to be potential predictors of the outcome. Since Complete Blood Count (CBC) is an inexpensive and routine test with a short turnaround time, finding an independent CBC factor to predict the severity of the disease would be of great value (5). Red Blood Cell Distribution Width (RDW) is one of the parameters of CBC and reflects the extent of anisocytosis/ heterogeneity of circulating erythrocytes volume (6). RDW has been reported to be increased in patients with alcohol abuse, hemolytic anemia, iron and B12/folate deficiency, and thrombotic and inflammatory conditions and can even be an independent factor predicting mortality in sepsis (7-12). Previous studies have shown that increased RDW is associated with mortality in patients with ARDS (13-15). A recent report has shown that the predictive capacity of RDW in predicting mortality is equal to Sepsis-related Organ Failure Assessment (SOFA) and Acute Physiology and Chronic Health Evaluation-II (APACHE-II) (16).

A meta-analysis found that the severely ill and expired COVID-19 patients had a significantly elevated RDW (17). Few studies have assessed the differential role of RDW in predicting the clinical outcomes and the prognosis of the illness in severe vs. moderate COVID-19 hospitalized patients (18-20). However, the association of RDW with adverse prognosis in COVID‐19 has not been well‐established. Besides, there is no consensus regarding the optimum predictive cut-off for RDW.

This study aimed to assess the possible relationship between RDW on admission and COVID-19 in-hospital mortality.

## 2. Methods

***2.1. Study design and setting***

This cross-sectional study was performed using the data of Isfahan COVID-19 Registry (I-CORE), a registry designated to COVID-19 patients who are admitted to hospitals of Isfahan, Iran. In I-CORE, demographic information, signs and symptoms of patients at the time of admission, laboratory data, and medications during the admission time are recorded. The design and methodology of I-CORE have been previously published in detail (21). The Ethics Committee of Isfahan University of Medical Sciences (IR.MUI.MED.REC.1399.1000) approved this study. Helsinki statement was observed throughout the study, and all data were managed anonymously.

*2.2. Participants*

Patients with a positive COVID-19 Reverse transcription-polymerase chain reaction (RT-PCR) test, who were older than 18 years and were registered on I-CORE from February 21st, 2020 to September 23rd, 2020 were included in this study. Patients without any RDW test on admission were excluded from the study.

*2.3. Measurements*

Age, sex (male, female), past medical history, O2 Saturation on admission (93% and higher, lower than 93%), RDW, White Blood Cell (WBC), Platelets (Plt), Red Blood Cell (RBC), Hemoglobin (Hb), Mean Corpuscular Volume (MCV) on admission, and ICU admission were extracted from the registry. Based on our current laboratory standards, RDW more than 14.5% was considered elevated. The age was considered both as a categorical and a continuous variable. Comorbidities were defined based on the International Statistical Classification of Disease and Related Health Problems (Tenth revision). 

Mortality during hospitalization was the primary outcome of the study. The severity of the disease was defined according to the New Coronavirus Pneumonia Prevention and Control Program (7th edition) published by the National Health Commission of China. Critical patients were defined as those with any of the following factors: (i) the need for mechanical ventilation in case of respiratory failure, (ii) shock, (iii) ICU admission due to simultaneous failure in another organ (22). 

**2.4. Statistical Analysis**

Data were analyzed using Statistical Package of Social Science software (SPSS version 18.0, IL, Chicago, USA). Mean ± standard deviation (SD) and frequency were used to report categorical and continuous variables, respectively. To compare the continuous and categorical variables between the survivor and non-survivor groups, independent t-test and chi-square were used, respectively. The Odds Ratio (OR) of mortality for elevated RDW was calculated in each age group.

Cox proportional hazards regression was used to calculate the mortality hazard ratio (HR). Variables with a significant association in the first analysis were entered as covariates in the final model. We performed a subgroup analysis to investigate the independent role of RDW as a predictive factor for mortality in severe and non-severe patients. A two-tailed P<0.05 was considered statistically significant. 

## 3. Results

*3.1 Patients’ baseline characteristics*

Data of 4152 patients were extracted from I-CORE and analyzed retrospectively. The mean age (±SD) of the patients was 61.1 (±16.97) years (56.2% male). Of 4152 patients, 477 (11.5%) expired during hospital admission. 597 (14.4%) cases were admitted to ICU, 272 (45.5%) of which expired. The mean age of the discharged patients was lower than the expired ones (p-value <0.001), but the difference between sex groups (male vs. female) was not statistically significant (p-value, 0.7). Table 1 compares some demographic, clinical, and laboratory parameters between survived and non-survived cases. 

*3.2. RDW and mortality risk*

The mean (±SD) RDW amongst survivors was lower than expired patients (13.9 ± 1.88 vs. 15.3 ± 2.7, respectively; p-value <0.0001). The difference between the mean RDW amongst discharged and expired patients was also found to be significant in all age groups (table 1). 

The mortality rate of COVID-19 patients who had an elevated RDW (greater than 14.5%) on admission was 21.2%, while those with normal RDW had a mortality rate of 7.8% (p-value <0.0001). The odds ratio of death for those with an elevated RDW was 3.1 (95% CI: 2.6-3.8). Table 2 compares the mortality rate between elevated and normal RDW stratified by age groups. The OR of mortality in all age groups was elevated significantly. The highest and lowest OR for mortality considering elevated RDW compared to normal RDW was in the group of younger than 50 years (4.4, 95% CI: 2.8-6.9) and in the group of 60-69 years (2.2, 95% CI: 1.4-3.5), respectively.

The HR of mortality for elevated RDW after adjustment for covariates was 2.03 (95% CI: 1.68 to 2.44, p-value < 0.0001; table 3). Also, ICU admission had a significant association with hospital mortality (HR: 7.1, 95% CI: 5.9 to 8.5, p-value <0.0001).

3.3. RDW and mortality risk considering the severity 

In the subgroup of severe disease, the result of cox proportional hazard regression analysis (Table 4.a and 4.b) showed a mortality HR of 1.47 (95% CI: 1.15-1.88; p-value: 0.002) for those with elevated RDW. Age was also associated with mortality in severe cases with an HR of 1.01 (95% CI: 1.007-1.024; p-value <0.0001).

**Figure 1:**
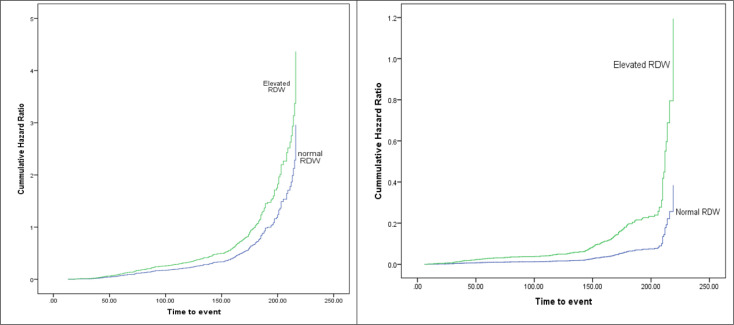
Mortality hazard ratio in patients with severe COVID-19 (left) and non-severe cases (right), in patients with elevated and normal red blood cell distribution width (RDW). Age, O2 saturation on admission, and having any comorbidities were entered as covariates. RDW more than 14.5 was defined as elevated; Event is defined as death; discharged patients were considered as censored

In the subgroup of non-severe patients, the HR of mortality for elevated RDW compared to normal RDW was 3.10 (95% CI: 2.35-4.09; p-value < 0.0001). The association between O2 saturation on admission and comorbidities with mortality was also significant with a hazard ratio of 1.9 (95% CI: 1.2- 3.0; P-value: 0.003) and 1.7 (95% CI: 1.1-2.4; p-value: 0.003), respectively. Figure 1 illustrates the cumulative hazard ratio for mortality in patients with elevated and normal RDW on admission in severe and non-severe patients, respectively. The cumulative HR for elevated RDW was higher than normal RDW, after adjustment for age, O2 saturation at admission, and having any comorbidity. This difference is more obvious in the group of non-severe patients.

**Table 1:** Comparing some demographic, clinical, and laboratory characteristics of survived and non-survived COVID-19 cases

**Variables**	**Mortality**		***p*** **-value**
	**No n = 3675**	**Yes n = 477 **	
**Age (year)**			
Mean ± SD	60.5 (16.81)	65.8 (17.47)	**<**0.0001
**Sex, N (%)**			0.7
Male	2063 (56.1)	271 (56.8)	
Female	1612 (43.9)	206 (43.2)	
**O2 saturation <93%, N (%)**			
On admission	2610 (71.0)	404 (84.7)	<0.0001
**RDW stratified by age group **			
Under 50	13.7 (1.90)	15.3 (2.60)	<0.0001
50-59	13.7 (1.80)	15.4 (3.19)	<0.0001
60-69	14.0 (1.92)	14.9 (2.56)	0.002
70-79	14.1 (1.80)	15.0 (2.77)	0.001
80 and higher	14.4 (1.91)	15.7 (2.60)	<0.0001
**Hematologic findings**			
WBC (×10*3/µL)	7.8 (4.82)	8.3 (7.27)	0.1
Plt (×10*3/µL)	196.1 (82.06)	203.5 (92.09)	0.06
RBC (×10*6/µL)	4.5 (0.75)	4.6 (0.72)	0.4
Hemoglobin (g/dl)	13.2 (2.22)	13.3 (2.29)	0.4
MCV	89.0 (7.60)	89.2 (7.39)	0.5
RDW	13.9 (1.88)	15.3 (2.73)	<0.0001
**Comorbidities, N (%)**			
Anemia	1253 (34.1)	158 (33.1)	0.6
Cancer	85 (2.3)	22 (4.6)	0.003
CVD	812 (22.3)	151 (31.8)	<0.0001
HTN	1207 (34.2)	199 (42.8)	<0.0001
Diabetes	1013 (27.9)	188 (39.6)	<0.0001
Immunodeficiency diseases	15 (0.4)	3 (0.6)	0.45
Non asthma Respiratory diseases	281 (7.7)	63 (13.3)	<0.0001
Asthma	98 (2.7)	10 (2.1)	0.45
At least one comorbidity	2154 (59.2)	376 (79.2)	<0.0001
**ICU admission, N (%)**	325 (8.8)	272 (57.0)	<0.0001

*Data were reported in mean (standard deviation; SD) or number (%).* *RDW: Red blood cell Distribution Width; WBC: White Blood Cells; Plt: Platelets; RBC: Red Blood Cells; MCV: Mean Corpuscular Volume; CVD: Cardiovascular Disease; HTN: Hypertension; ICU: Intensive Care Unit; P-value <0.05 is considered statistically significant. *

**Table 2:** Relation between mortality Rate (%) and elevated Red Blood cell Distribution Width (RDW) Stratified by Age groups

**Age (year)**	**Normal RDW**		**Elevated RDW** ^*^		**OR** ^†^ ** (95% CI** ^‡^ **) **	**P-value**
	**Total**	**death**	**Total**	**death**		
< 50	841	44 (5.2)	238	47 (19.7)	4.4 (2.8-6.9)	<0.0001
50-59	601	34 (5.7)	168	31 (18.5)	3.7 (2.2-6.3)	<0.0001
60-69	649	51 (7.9)	224	36 (16.1)	2.2 (1.4-3.5)	<0.0001
70-79	532	57 (10.7)	238	53 (22.3)	2.3 (1.5-3.6)	<0.0001
≥ 80	390	49 (12.6)	271	75 (27.7)	2.6 (1.7-3.9)	<0.0001
Total	3013	235 (7.8)	1139	242 (21.2)	3.1 (2.6-3.8)	<0.0001

Elevated RDW is considered as the RDW more than 14.5% on admission; Data are presented as number (%).^ *^RDW: Red Blood cell Distribution Width; ^†^OR: Odds Ratio of mortality; ^‡^CI: Confidence interval.

**Table 3:** Cox multivariate proportional hazard regression model fitted to the inpatient mortality of COVID-19 patients

**Variables**	**b**	**SE (b)**	**Wald**	**P**	**HR** ^†^ **= Exp (b)**	**95.0% CI **	
						**Lower**	**Upper**
Age (years)	0.01	0.003	14.8	<0.0001	1.012	1.006	1.018
RDW^*^ (>14.5%)	0.71	0.095	56.04	<0.0001	2.033	1.689	2.448
ICU (Yes)	1.96	.096	421.35	<0.0001	7.122	5.905	8.590
O2 sat (< 93%)	0.07	0.13	0.32	0.56	1.077	0.835	1.391
Comorbidity(Yes)	0.16	0.12	1.96	0.161	1.183	0.935	1.497

^*^ **RDW: Red Blood cell Distribution Width;** ^†^ **HR: hazard Ratio; ICU: Intensive care unit; CI: confidence interval; O2 sat: O2 saturation. **

**Table 4a:** Cox multivariate proportional hazard regression model fitted to the inpatient mortality of severe COVID-19 patients

**Variables**	**b**	**SE(b)**	**Wald**	**P**	**HR= Exp(b)**	**95.0% CI **	
						**Lower**	**Upper**
Age (years)	0.015	0.004	14.32	<0.0001	1.016	1.007	1.024
RDW (>14.5%)	0.38	0.12	9.59	0.002	1.47	1.15	1.88
O2 sat (<93%)	-0.28	0.16	3.16	0.07	0.75	0.54	1.03
Comorbidity(Yes)	-0.10	0.15	0.46	0.49	0.89	0.65	1.22

**RDW: Red Blood cell Distribution Width; HR: hazard Ratio; ICU: Intensive care unit; CI: confidence interval; O2 sat: O2 saturation. **

**Table 4b:** Cox multivariate proportional hazard regression model fitted to the inpatient mortality of non-severe COVID-19 patients

**Variables**	**b**	**SE(b)**	**Wald**	p	**HR= Exp(b)**	**95.0% CI **	
						**Lower**	**Upper**
Age (years)	0.003	0.005	0.55	0.45	1.003	0.994	1.013
RDW (>14.5%)	1.13	0.14	64.14	<0.0001	3.10	2.35	4.09
O2 sat (<93%)	0.66	0.22	8.77	0.003	1.94	1.25	3.02
Comorbidity(Yes)	0.53	0.18	8.67	0.003	1.70	1.19	2.42

**RDW:** **Red Blood cell Distribution Width** **; HR: hazard Ratio; ICU: Intensive care unit; CI: confidence interval; O2 sat: O2 saturation. **

## 4.Discussion

Identifying patients at highest risk for severe disease is essential to facilitating early, aggressive intervention and managing local hospital resources to mitigate the critical care crises. In the current study, the possible association between elevated RDW on admission and mortality risk in COVID-19 patients was assessed and as assumed, elevated RDW was associated with mortality in COVID-19 patients.

Compared to other markers with a significant prognostic value in SARS-CoV2 infection, RDW has some advantages because of its capability to efficiently predict the risk of mortality in the general population and patients with sepsis, ARDS, pneumonia, and other respiratory tract infections (23-26). 

Several pathophysiological mechanisms affect RBCs homeostasis and lead to an increase in RDW. Hypoxemia associated with COVID-19 is one of them, which induces erythropoietin (EPO) release, as reported in several similar lung diseases (27, 28). The EPO increases RBC formation rate and RBC volume, which increase RDW (29). Inflammatory cytokines associated with inflammatory diseases also increase RDW (30, 31). Likewise, inflammation can slow down the maturation of RBCs, leading to reticulocytosis and an increase in RDW (32). Another possible mechanism could be an overstimulation of the bone marrow following COVID‐19 infection, which impacts RBC production, resulting in a broader range of RBC size and thus, elevated RDW levels (33).

As shown by the results of our study, an elevated RDW at the time of admission in COVID-19 patients is associated with a higher mortality rate (21.2% in patients with elevated RDW compared to 7.8% in patients with normal RDW) in all age groups. In a similar study by Foy BH et al. on 1641 COVID-19 patients, RDW greater than 14.5% was associated with a higher mortality rate (18); also, results of a study by Hornick et al. show that each 1% increase in RDW is associated with a 39% increase in the mortality rate (34). As Wang et al. stated, RDW was a prognostic predictor of illness severity in 98 COVID-19 patients (35). In a prospective observational study on 143 cases, Lorente et al. declared that non-surviving COVID-19 patients had higher RDW on ICU admission with a higher mortality rate showing that RDW values greater than 13% have good performance in prediction of 30-day mortality (16). The results of our study show that an elevated RDW (more than 14.5%) has a mortality hazard ratio of 1.47 in comparison to normal RDW. In a retrospective study on 225 hospitalized COVID-19 patients in Iran, RDW at the time of admission was neither related to mortality nor to ICU admission; which can be of lower value compared to the results of our article since the sample population was larger in our study (36). Our study also showed that elevated RDW is a stronger predictor of mortality in moderate COVID-19 hospitalized patients compared to severe/critical cases. This may be due to the effect of different factors on RDW such as comorbidities, anemia, age, and inflammatory state. In line with our results, Han et al. showed that RDW was able to predict all-cause mortality in those with critical illness, independent from severity scores (37). It is worth mentioning that RDW is usually a slow changing measure (18), so large increase in the non-severe patient group may propose a longer duration of disease for these patients at the time of admission, but accurate measurement in the earlier phases of the disease is required to test this assumption.

As for other assessed parameters besides RDW elevation, modeling shows a negligible association between age and mortality with an HR of 1.01, while the association of elevated RDW with mortality is obvious in all age groups. As reported in a meta-analysis, the most significant increase in mortality risk was observed in patients aged 60 to 69 years (38). In contrast to age, patients’ sex did not seem to affect the mortality in our study; yet, in a cohort of 200 hospitalized patients with COVID-19, male sex was associated with increased oxygenation requirements, higher in-hospital mortality, and worse hospital outcomes (39). Other studies have also identified male sex as a risk factor for worse outcomes and increased mortality (40, 41). O2 saturation status on admission is another parameter associated with the mortality rate in COVID-19 patients. In an observational study by Jain et al., oxygen saturation below 93% (with or without supplemental support) was known as an important early marker or predictor of in-hospital death (OR= 17.68) (42). The results of our study show a weak association between O2 saturation of less than 93% with mortality in non-severe patients; this is while it had no significant association with mortality in ICU patients. This difference could be due to the level of O2 saturation, which is much lower in all ICU patients, and the cut-off of 93% could not differentiate poor outcome patients. Previous reports have shown a correlation between RDW and inflammatory cytokines such as tumor necrosis factor (TNF)-alpha, interleukin (IL)-1 and IL-6, and between RDW and oxidative stress (1616). It can be speculated that the association between RDW and comorbid disorder in our study could be due to a higher degree of inflammation and oxidative stress in patients with cardiovascular disease, diabetes, cancer, and hypertension.

RDW is a valuable laboratory parameter for predicting mortality, especially in patients without ICU admission. It is suggested to check RDW on admission and consider its elevation as a predictor of poor outcomes. This indicator could be used as a criterion to prioritize patients for early and aggressive interventions, and better management of hospital resources.

## 5. Limitations

The main point of this study was to evaluate the potential value of RDW in risk stratification of COVID-19 admitted patients in a large population based on a local COVID-19 registry. Since the analysis was limited to hospitalized patients, the results may not be applied to non-hospitalized infected individuals. Moreover, the absence of D-dimer and BMI in our data is another limitation of our study. RDW changes were not followed during patients’ hospitalization, which is another limitation in this article. Regarding all stated limitations, more studies are needed in the field of the current study. 

## 6. Conclusion

The results support the presence of an association between elevated RDW and mortality in patients with COVID-19, especially those who were not admitted to ICU. It seems that elevated RDW could be used as a predictor of mortality in COVID-19 cases.

## 7. Declarations

### 7.1. Acknowledgments

The authors wish to thank Mrs. Azam Mosayebi for her assistance during data gathering and other associated healthcare providers and hospital staff for their selfless services and cooperations during COVID-19. 

### 7.2. Conflict of interest

The authors declare no conflict of interest.

### 7.3. Funding

This research was supported by the Isfahan University of Medical Sciences.

### 7.4. Authors’ contributions

SH, GV, AV, and SJ performed the study concept and design. AM was involved in data acquisition. AV and MN performed the statistical analysis. SH, AV, and SJ performed data analysis and interpretation. SH, AV, SJ, and AM wrote the first draft of the manuscript. All authors contributed to the critical revision and final approval of this manuscript. 

### 7.5. Data Presentation

The information of this manuscript has not been presented in any meeting(s).
